# Maternal interpregnancy weight change and premature birth: Findings from an English population-based cohort study

**DOI:** 10.1371/journal.pone.0225400

**Published:** 2019-11-21

**Authors:** Grace Grove, Nida Ziauddeen, Scott Harris, Nisreen A. Alwan

**Affiliations:** 1 School of Primary Care, Population Sciences and Medical Education, Faculty of Medicine, University of Southampton, Southampton, United Kingdom; 2 NIHR Southampton Biomedical Research Centre, University of Southampton and University Hospital Southampton NHS Foundation Trust, Southampton, United Kingdom; University of Botswana, BOTSWANA

## Abstract

**Background:**

The relationship between maternal weight change between pregnancies and premature birth is unclear. This study aimed to investigate whether interpregnancy weight change between first and second, or second and third pregnancy is associated with premature birth.

**Methods:**

Routinely collected data from 2003 to 2018 from one English maternity centre was used to produce two cohorts. The primary cohort (n = 14,961 women) consisted of first and second live-birth pregnancies. The secondary cohort (n = 5,108 women) consisted of second and third live-birth pregnancies. Logistic regression models were used to examine associations between interpregnancy BMI change and premature births adjusted for confounders. Subgroup analyses were carried out, stratifying by initial pregnancy BMI groups and analysing spontaneous and indicated premature births separately.

**Results:**

In the primary cohort, 3.4% (n = 514) of births were premature compared to 4.2% (n = 212) in the secondary cohort, with fewer indicated than spontaneous premature births in both cohorts.

**Primary cohort:**

Weight loss (>3kg/m^2^) was associated with increased odds of premature birth (adjusted odds ratio (aOR):3.50, 95% CI: 1.78–6.88), and spontaneous premature birth (aOR: 3.34, 95%CI: 1.60–6.98), in women who were normal weight (BMI 18.5-25kg/m^2^) at first pregnancy. Weight gain >1kg/m^2^ was not associated with premature birth regardless of starting BMI.

**Secondary cohort:**

Losing >3kg/m^2^ was associated with increased odds of premature birth (aOR: 2.01, 95%CI: 1.05–3.87), when analysing the whole sample, but not when restricting the analysis to women who were overweight or obese at second pregnancy.

**Conclusions:**

Normal-weight women who lose significant weight (>3kg/m^2^) between their first and second live pregnancies have greater odds of premature birth compared to normal-weight women who remain weight stable in the interpregnancy period. There was no evidence of association between weight change in women who were overweight or obese at the start of their first pregnancy and premature birth.

## Introduction

In 2015, premature birth (PB) (before 37 completed weeks of gestation) [1] was the leading cause of death in children under five years old, responsible for 1,005,000 deaths worldwide [2]. In the UK, approximately 7.8% of livebirths are premature [3]. Those who survive may face a large burden of morbidity and ongoing healthcare needs [4–6]. Obesity is a pressing health concern worldwide [7], and maternal obesity is associated with numerous adverse effects [8]. In England, overweight and obesity is common in women of reproductive age, with 37% of 16–24 year old women, 57% of 25–34 year old women and 65% of 35–44 year old women being classified as overweight or obese in 2017 [9]. Maternal weight has been linked to both spontaneous (low maternal weight) and indicated (high maternal weight) PB [10]. Recently, PB has also been linked with maternal interpregnancy weight change (weight change between the first antenatal (booking) appointment for one pregnancy and the booking appointment for the next pregnancy) [11–13]. However, there is a lack of consistency from the evidence, with other studies finding no association [14, 15]. Additionally, the available evidence only considers first to second pregnancy, with the exception of Wallace et al [15], who examined the relationship across the first three pregnancies and found no association.

The mechanisms that may underlie these associations are poorly understood, but may include maternal undernutrition [16] and maternal infection and inflammation [10, 16]. Maternal undernutrition may also contribute to maternal infection [16, 17], if the women is deficient in key micronutrients for immune responses. Other possible mechanisms include poor placental function, in which maternal undernutrition may also play a role [13]. Finally, obesity related co-morbidities such as diabetes and pre-eclampsia [12], and stress [17] may also contribute to increasing the risk of PB. It is possible that the associations seen are not true associations, but instead are markers for unmeasured confounding factors that are causing weight change, and it is these factors, not the weight change, that are actually associated with PB. For example, psychosocial stress and underlying poor mental and physical health may all be contributing to both weight loss [18] and PB [17].

This study aimed to examine the association between maternal interpregnancy weight change and PB (overall, spontaneous and indicated), in a population-based cohort in the South of England, both between first and second, and second and third pregnancies.

## Methods

### Study sample

Routinely collected maternity healthcare data from a population-based cohort registered at the University Hospital Southampton (UHS) National Health Service (NHS) Trust, UK (2003–2018) were used (a total of 96,489 pregnancies). The database included anonymised antenatal care and birth data for each pregnancy within the study time period, and hence included more than one pregnancy for some of the mothers. This analysis forms part of a research project (SLOPE) approved by the University of Southampton Faculty of Medicine Ethics Committee (ID 24433) and the National Health Service Health Research Authority (IRAS 242031).

#### Inclusion and exclusion criteria

This analysis included women who have their first and second or second and third consecutive live-born singleton pregnancies within the dataset. Women missing key data (weight or height at booking, or gestation at birth), or who had inconsistent or outlying values for key data that were likely to be errors were excluded from the analysis. In order to mitigate for inaccurate self-reported heights, women who lost height between pregnancies (>5cm), and those who gained height (>5 cm and were over the age of 22 at first pregnancy), were excluded from the analysis. Women who were booked after 24 weeks gestation were excluded, as the study centre is a specialist centre, and some of these women booking later may have been high risk pregnancies referred from another unit. Multiple births were excluded due to their strong association with PB [10]. Women with multiple miscarriages were also excluded as these women are likely to have different risk factors to the general population [19]. Variables that should have remained constant between pregnancies (e.g. ethnicity) were checked for agreement and noted as missing if there were improbable changes, to prevent incorrect variables from being entered into the analysis.

**Fig 1:**
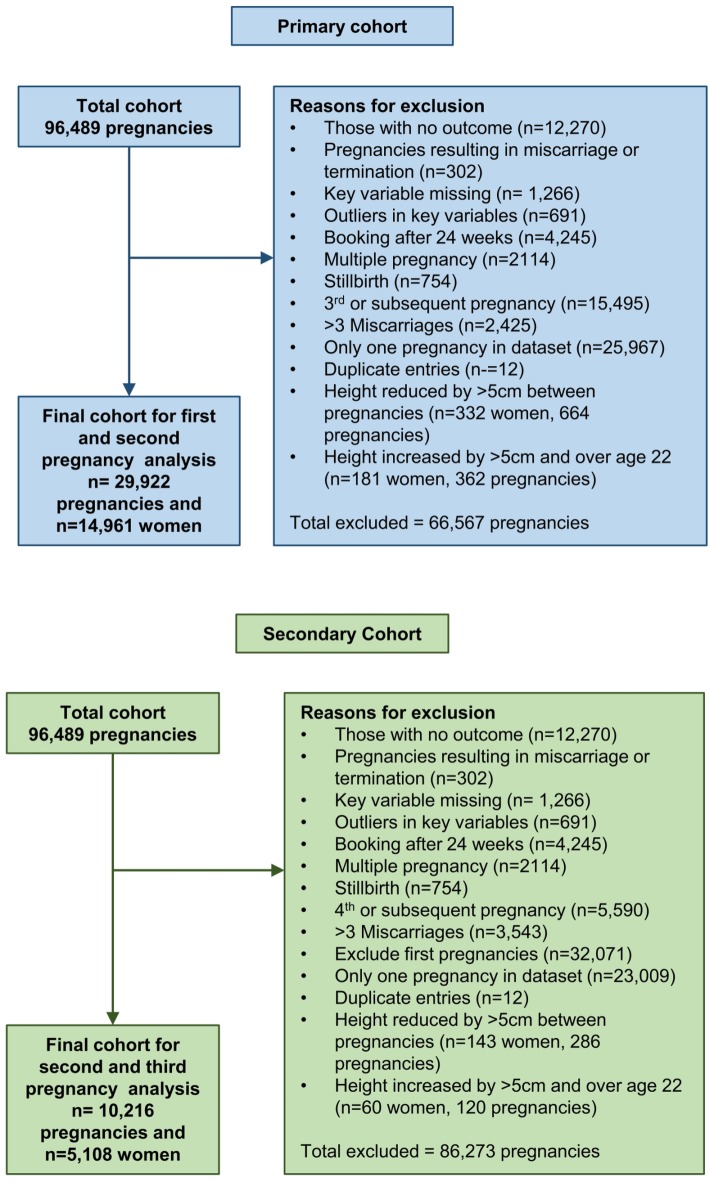
Flow diagram depicting data preparation process for all analyses on women with consecutive first and second (primary cohort) or second and third (secondary cohort) live-born infants in a population-based cohort from Southampton, UK from 2003 to 2018.

Once the data had been prepared (Fig 1) the final sample size for first and second consecutive live-births (the primary cohort) was 29,922 pregnancies, which equated to 14,961 women. The final sample size for second and third consecutive live-births (the secondary cohort) was 5,108 women (10,216 pregnancies).

### Assessment of exposure

Maternal height (m) was self-reported, maternal weight (kg) was measured at booking, and both were recorded at the first antenatal (booking) appointment for each pregnancy. Weight was measured in light clothing, with a single measurement being taken and recorded by the midwife. Women whose height was inconsistent between pregnancies were excluded, with the exception of those changes likely to be due to maternal growth. The maternal booking height and weight were used to calculate body mass index (BMI) and BMI group (Underweight: <18.5 kg/m^2^, normal weight: 18.5 to <25 kg/m^2^, overweight: 25 to <30 kg/m^2^ and obese: ≥30 kg/m^2^) [20].

For the purposes of this study, and in keeping with the literature [11–13], interpregnancy weight change was defined as a change in BMI between the booking appointment for one live-birth pregnancy and the booking appointment for the next consecutive live-birth pregnancy. In the UK, it is recommended that the booking appointment takes place by 10 weeks [21]. BMI at booking was compared between pregnancies, and change categories were assigned (loss: >3 kg/m^2^, loss: >1 to ≤3 kg/m^2^, weight stable: ≤1 BMI kg/m^2^, weight gain: >1 to ≤3 kg/m^2^ and weight gain >3 kg/m^2^). There is no consensus in the literature as to appropriate categories for this type of analysis. Some studies used change in BMI categories [22, 23] whilst others used change in BMI value stratified into various categories ranging from 1 kg/m^2^ to 5kg/m^2^. However, several other studies have used similar categorisation to this study [13, 24, 25], and it was felt that these categories would be discriminating enough to differentiate between those who gained or lost a relatively small amount of weight and those who were stable or experienced large changes in weight, while allowing the study to maintain power in the sub analyses.

### Assessment of outcome

Gestation at delivery was available for each pregnancy, and it was assumed that, in the vast majority of women, this was based on a dating ultrasound scan (USS), as recommended by the National Institute for Health and Care Excellence (NICE) [21]. Gestation at delivery was used to create a categorical outcome variable, indicating if the infant was premature or not (<37 weeks gestation). A further outcome variable indicating whether the delivery was spontaneous or induced was also created, using the following variables: whether the woman was induced or not and type of delivery, for example planned C-sections were coded as indicated deliveries.

### Assessment of covariates

Measured confounders and competing exposures were adjusted for within the analysis. Self-reported maternal factors adjusted for (all except previous PB at the later pregnancy) included ethnicity [10, 26–29], highest educational achievement (school or college, higher education or unknown) [30–33], smoking status [34–36], employment status [26, 37–39] and previous PB [40]. Other factors adjusted for included age [26, 41–43], baseline BMI and gestational age at time of measurement (at the start of the initial pregnancy) [44], co-morbidities [45], the use of fertility treatment [46, 47], infant gender [48], pregnancy complications [45] and interpregnancy interval [49, 50]. Interpregnancy interval was calculated using date of delivery for both infants, then subtracting the gestational age at birth for the second infant, to give the time span from one live delivery to the conception of the next pregnancy resulting in a live birth [51]. Co-morbidities included diabetes and hypertension, and pregnancy complications included pre-eclampsia, eclampsia, gestational hypertension, gestational diabetes and HELLP (haemolysis, elevated liver enzymes and low platelet count).

The alcohol intake variable was self-reported and the way the electronic system was set up allowed for ‘no answer’ to be potentially recorded as ‘no’. The percentage of women reporting drinking any alcohol at their booking appointment (5%) was substantially lower than has been reported in the literature (25% [52] to 28.5% [53] for the UK). For this reason alcohol consumption was not included in any modelling as this variable was deemed unreliable.

### Statistical analysis

Statistical analysis was undertaken in Stata version 15.0SE [54]. When comparing parametric continuous descriptive information looking for differences by both exposure and outcome, either an independent samples t-test or oneway ANOVA was used. When looking at non-parametric continuous data, a Mann-Whitney U test or Kruskal-Wallis test was used. When performing analysis on categorical descriptive variables, a Chi-square or Fisher’s exact test was used to look for significant differences. Logistic regression was used to model the association of interpregnancy weight change with PB. This model was built up by adding in a priori selected covariates in stages until all available variables hypothesised to affect the relationship had been adjusted for. Subgroup analyses were then undertaken as follows:

- Analysis stratified by starting BMI category at initial (first in primary cohort, second in secondary cohort) pregnancy
- Comparing spontaneous PBs with term births (excluding indicated PBs)
- Comparing indicated PBs with term births (excluding spontaneous PBs)

## Results

**Fig 2:**
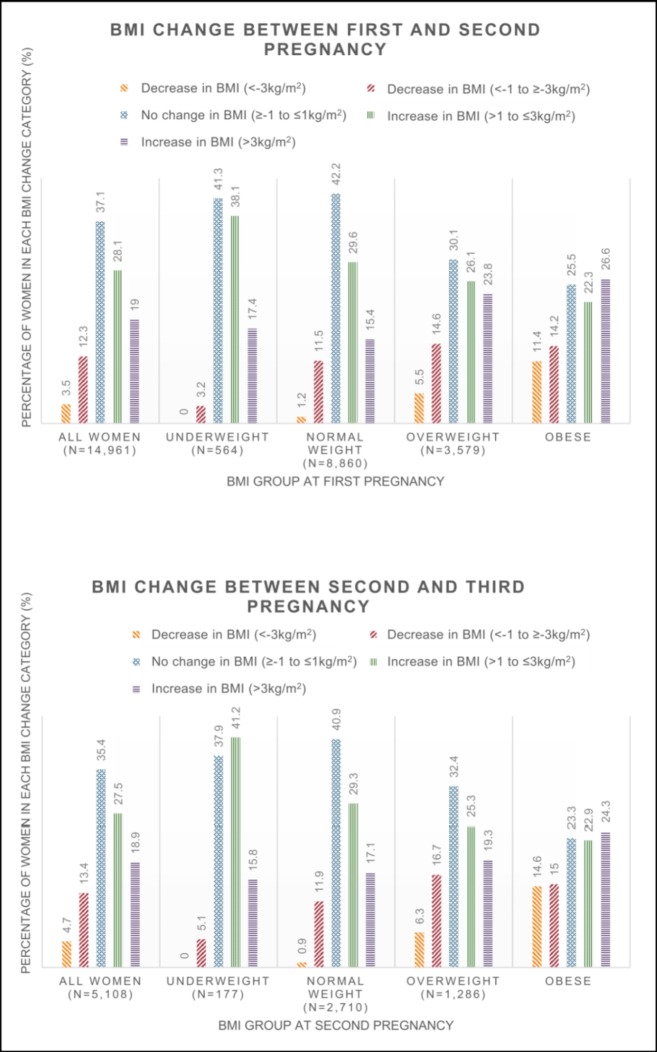
The percentage of women in each change in BMI category for both the primary (first to second pregnancy) and secondary (second to third pregnancy) cohorts, subdivided by BMI category at initial pregnancy in a population-based cohort from Southampton, UK from 2003 to 2018.

The median change in early pregnancy BMI between first and second live pregnancies (primary cohort) was a gain of 0.8 kg/m^2^ (IQR -0.4 to 2.4 kg/m^2^) (Fig 2). Overall, 37.1% of women stayed weight stable, 47.1% gained weight (>1 kg/m^2^) and 15.8% lost weight (>1 kg/m^2^). Of those who gained weight (>1 kg/m^2^), the median BMI increase was 2.52 kg/m^2^ (IQR 1.64–3.95 kg/m^2^). Of those who lost weight (>1 kg/m^2^), the median BMI decrease was 1.86 kg/m^2^ (IQR 1.33–2.83 kg/m^2^).

The median change in early pregnancy BMI between second and third live pregnancies (secondary cohort) was a gain of 0.8 kg/m^2^ (IQR -0.5 to 2.4 kg/m^2^). Overall, 35.4% of women stayed weight stable, 46.5% gained weight (>1 kg/m^2^) and 18.1% lost weight (>1 kg/m^2^). Of those who gained weight (>1 kg/m^2^), the median BMI increase was 2.55 kg/m^2^ (IQR 1.68–3.91 kg/m^2^). Of those who lost weight (>1 kg/m^2^), the median BMI decrease was 2.03 kg/m^2^ (IQR 1.37–3.06 kg/m^2^).

Women who were weight stable (≤1 kg/m^2^ weight change) in both cohorts were on average older than their counterparts, had a lower starting BMI, and were more likely to have a university degree and be non-smokers.

**Fig 3:**
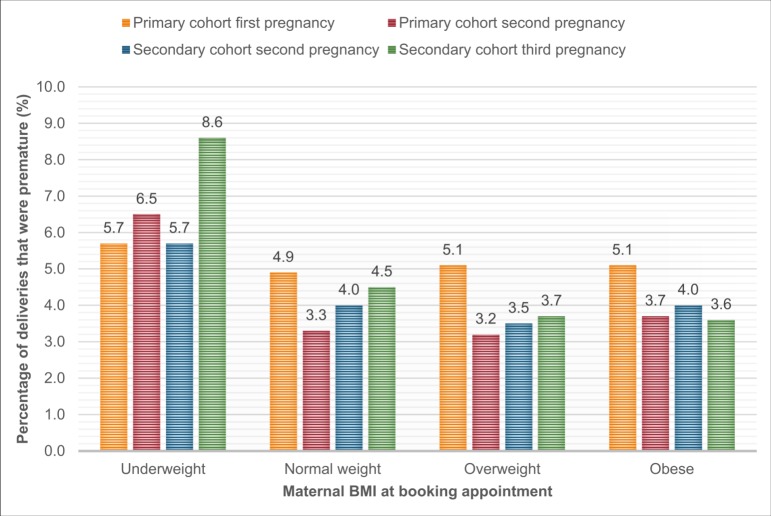
The percentage of live births that were premature for each pregnancy (first, second or third), by maternal body mass index (BMI) at the first antenatal appointment.

In the primary cohort, 3.4% (n = 514) of second pregnancy births were premature compared to 4.2% (n = 212) of third pregnancy births in the secondary cohort. There was a higher percentage of PBs in women who were underweight at booking than those who were not (Fig 3).There were fewer indicated than spontaneous PBs in both cohorts (primary cohort, 16.9% of PBs were indicated, secondary cohort 18.4% of PBs were indicated). Women who had a PB were more likely to be smokers and to have comorbidities, and were less likely to have a university degree (Table 1, Table 2).

**Table 1:** Characteristics of mothers and infants born to second live birth pregnancy, categorised by whether the birth was premature (<37 weeks gestation) in a population-based cohort from Southampton, UK from 2003 to 2018.

		Primary cohort- outcome of second pregnancy				P value
		Term birth		Premature birth		
**N (% of cohort)**		14,447	96.6	514	3.4	
**Maternal age, years (median, IQR)**		**29**	**25–33**	**29**	**23–33**	**0.006**^$^
**Maternal BMI at initial pregnancy, kg/m**^**2**^ **(median, IQR)**		23.5	21.2.-26.9	23.7	20.8–27.2	0.485^$^
**Maternal BMI at subsequent pregnancy, kg/m**^**2**^ **(median, IQR)**		24.5	21.9–28.5	24.4	21.4–28.7	0.275^$^
**Maternal BMI category at initial pregnancy, kg/m**^**2**^ **(%, 95% CI)**	**Underweight <18.5**	**3.6**	**3.3–3.9**	**7.6**	**5.5–10.2**	**<0.001**^**#**^
	**Normal weight 18.5–24.9**	**59.4**	**58.6–60.2**	**55.6**	**51.2–60.0**	
	**Overweight 25.0–29.9**	**24.0**	**23.3–24.7**	**21.4**	**17.9–25.2**	
	**Obese ≥30.0**	**13.0**	**12.5–13.6**	**15.4**	**12.4–18.8**	
**Interpregnancy interval, months (median, IQR)**		22.7	14.6–34.8	24.6	14.0–40.3	0.106^$^
**Maternal ethnicity (%, 95% CI)**	**White**	86.9	86.3–87.4	86.2	82.9–89.1	0.280^
	**Other**	13.2	12.6–13.7	13.8	10.9–17.1	
**Maternal education attainment, (%, 95% CI)**	**College or lower**	**61.4**	**60.6–62.2**	**69.8**	**65.7–73.8**	**<0.001**^**#**^
	**Higher education**	**35.2**	**34.4–36.0**	**25.1**	**21.4–29.1**	
	**Unknown**	**3.4**	**3.1–3.7**	**5.1**	**3.3–7.3**	
**Maternal employment (%, 95% CI)**	**Employed**	**68.1**	**66.9–68.4**	**62.9**	**58.3–66.8**	**0.013**^**#**^
	**Unemployed**	**31.9**	**30.9–32.4**	**37.1**	**32.8–41.3**	
**Maternal smoking status (%, 95% CI)**	**Non-smoker (including ex-smokers)**	**87.7**	**87.1–88.2**	**79.2**	**75.4–82.6**	**<0.001**^**#**^
	**Current smoker**	**12.3**	**11.8–12.9**	**20.8**	**17.4–24.6**	
**Gestation at booking appointment for second pregnancy, days (mean, SD)**		77.0	16.4	75.7	18.5	0.078*
**Prematurity category of previous pregnancy (%, 95% CI)**	**Extremely premature**	**0.2**	**0.1–0.3**	**2.0**	**0.9–3.5**	**<0.001**^**^**^
	**Very premature**	**0.3**	**0.2–0.4**	**4.7**	**3.0–6.9**	
	**Moderate to late premature**	**3.7**	**3.3–4.0**	**22.4**	**18.8–26.2**	
	**Term**	**95.9**	**95.5–96.2**	**71.0**	**66.9–74.9**	
**Mode of birth category (%, 95% CI)**	**Spontaneous labour**	**76.5**	**75.8–77.2**	**83.1**	**79.5–86.2**	**0.001**^**#**^
	**Induced delivery**	**23.5**	**22.8–24.2**	**16.9**	**13.8–20.5**	
**Fertility treatment (%, 95% CI)**	**Yes**	1.6	1.4–1.8	2.1	1.0–3.8	0.318^**#**^
**Comorbidities (%, 95% CI)**	**Yes**	**7.0**	**6.5–7.4**	**17.1**	**14.0–20.7**	**<0.001**^**#**^
**Gender of infant (%, 95% CI)**	**Male**	**51.3**	**50.4–52.1**	**59.3**	**54.8–63.4**	**<0.001**^**#**^

*Tested for significance using an independent samples T-Test

^#^Tested for significance using Chi squared test

^$^Tested for significance using Mann Whitney-U test

^^^Tested for significance using Fisher’s exact test

Significant P values in bold

**Table 2:** Characteristics of mothers and infants born to third live birth pregnancy, categorised by whether the birth was premature (<37 weeks gestation) in a population-based cohort from Southampton, UK from 2003 to 2018.

		Secondary cohort- outcome of third pregnancy				P value
		Term birth		Premature birth		
**N (% of cohort)**		4,896	95.9	212	4.2	
**Maternal age, years (median, IQR)**		29	26–33	29	24–33	0.068*
**Maternal BMI at initial pregnancy, kg/m**^**2**^ **(median, IQR)**		**24.2**	**21.6–28.3**	**23.9**	**20.7–27.8**	**0.038**^**$**^
**Maternal BMI at subsequent pregnancy, kg/m**^**2**^ **(median, IQR)**		25.3	22.3–29.5	24.7	22.0–28.4	0.087^**$**^
**Maternal BMI category at initial pregnancy, kg/m**^**2**^ **(%, 95% CI)**	**Underweight <18.5**	3.4	2.9–3.9	5.7	3.0–9.7	0.276^#^
	**Normal weight 18.5–24.9**	53.0	51.6–54.4	54.3	47.3–61.1	
	**Overweight 25.0–29.9**	25.2	24.0–26.5	24.1	18.5–30.4	
	**Obese ≥30.0**	18.4	17.3–19.5	16.0	11.4–21.7	
**Interpregnancy interval, months (median, IQR)**		24.8	14.1–42.6	24.9	12.5–47.4	0.907^**$**^
**Maternal ethnicity (%, 95% CI)**	**White**	82.4	81.3–83.4	85.4	79.9–89.8	0.822^
	**Other**	17.6	16.6–18.7	14.6	10.2–20.1	
**Maternal education attainment, (%, 95% CI)**	**College or lower**	75.3	74.0–76.4	79.7	73.7–84.9	0.162^#^
	**Higher education**	22.3	21.1–23.5	17.0	12.2–22.7	
	**Unknown**	2.5	2.1–3.0	3.3	1.3–6.7	
**Maternal employment (%, 95% CI)**	**Employed**	49.2	47.4–50.2	44.0	36.6–50.4	0.144^#^
	**Unemployed**	50.8	50.0–51.8	56.0	48.2–62.0	
**Maternal smoking status (%, 95% CI)**	**Non-smoker (including ex-smokers)**	**80.1**	**80.0–81.2**	**67.9**	**61.2–74.2**	**<0.001**^#^
	**Current smoker**	**19.9**	**18.8–21.0**	**32.1**	**25.8–38.8**	
**Gestation at booking appointment for second pregnancy, days (mean, SD)**		79.5	18.5	81.6	21.8	0.120*
**Prematurity category of previous pregnancy (%, 95% CI)**	**Extremely premature**	**0.3**	**0.2–0.5**	**0.9**	**0.1–3.4**	**<0.001**^
	**Very premature**	**0.2**	**0.1–0.3**	**1.4**	**0.3–4.1**	
	**Moderate to late premature**	**2.7**	**2.2–3.2**	**19.8**	**14.7–25.8**	
	**Term**	**96.9**	**96.4–97.4**	**77.8**	**71.6–83.2**	
**Delivery category (%, 95% CI)**	**Spontaneous labour**	**73.8**	**72.6–75.1**	**81.6**	**75.7–86.6**	**0.011**^#^
	**Induced delivery**	**26.1**	**24.9–27.4**	**18.4**	**13.4–24.3**	
**Fertility treatment (%, 95% CI)**	**Yes**	0.9	0.7–1.2	0.9	0.1–3.4	0.716^
**Comorbidities (%, 95% CI)**	**Yes**	**7.5**	**6.8–8.2**	**18.4**	**13.4–24.3**	**<0.001**^#^
**Gender of infant (%, 95% CI)**	**Male**	50.7	49.2–52.1	53.8	46.3–60.2	0.370^#^

*Tested for significance using an independent samples T-Test

^#^Tested for significance using Chi squared test

^$^Tested for significance using Mann Whitney-U test

^^^Tested for significance using Fisher’s exact test

Significant P values in bold

There was no significant association found between interpregnancy weight change between first and second pregnancy and PB in the whole primary cohort sample in both the univariable and the multivariable logistic regression models (Table 3). When considering the association between weight change from second to third pregnancy and PB, weight loss (>3 kg/m^2^) between second and third pregnancy was found to be associated with an increased odds of PB in the whole secondary cohort sample using a fully adjusted model (adjusted odds ratio (aOR) 2.01, 95% CI 1.05–3.87) (Table 4).

**Table 3:** Associations of weight change between first and second live-birth pregnancies with PB at second pregnancy, stratified by BMI group at the start of the first pregnancy, in a population-based cohort. Princess Anne Hospital, Southampton, UK 2003–2018.

Model	BMI group at first pregnancy (n)	Decrease in BMI (<-3 units)			Decrease in BMI (<-1 to ≥ -3 units)			Increase in BMI (>1 to ≤3 units)			Increase in BMI (>3 units)		
		OR	95% CI	P value	OR	95% CI	P value	OR	95% CI	P value	OR	95% CI	P value
**Unadjusted**	**All women (14,961)**	1.39	0.90–2.14	0.141	1.16	0.87–1.53	0.311	0.94	0.75–1.19	0.619	1.11	0.87–1.42	0.403
	Underweight (564)**	-	-	-	0.59	0.75–4.68	0.621	0.54	0.26–1.16	0.114	0.66	0.26–1.68	0.383
	**Normal weight (8,860)**	***4*.*00***	***2*.*15–7*.*58***	***<0*.*001***	1.31	0.90–1.90	0.153	0.97	0.72–1.30	0.844	1.25	0.89–1.76	0.191
	**Overweight (3,579)**	1.11	0.49–2.53	0.807	1.00	0.56–1.81	0.989	0.92	0.56–1.52	0.746	0.83	0.48–1.40	0.483
	**Obese (1,958)**	0.61	0.22–1.67	0.337	1.32	0.64–2.73	0.457	1.15	0.60–2.25	0.675	1.35	0.73–2.51	0.342
Fully-adjusted*	**All women (14,845)**	1.34	0.84–2.13	0.220	1.16	0.87–1.56	0.311	0.89	0.71–1.13	0.347	0.91	0.70–1.19	0.486
	Underweight (555)**	-	-	-	0.42	0.05–3.93	0.446	0.45	0.19–1.04	0.062	0.52	0.18–1.49	0.235
	**Normal weight (8,788)**	***3*.*50***	***1*.*78–6*.*88***	***<0*.*001***	1.25	0.85–1.84	0.259	0.94	0.69–1.28	0.683	0.98	0.68–1.42	0.921
	**Overweight (3,552)**	1.14	0.48–2.71	0.764	1.06	0.57–1.95	0.858	0.90	0.53–1.52	0.693	0.76	0.43–1.35	0.354
	**Obese (1,945)**	0.57	0.20–1.62	0.288	1.48	0.69–3.18	0.319	1.14	0.56–2.29	0.721	1.20	0.62–2.35	0.590

Odds ratios (OR), confidence intervals (CI) and P value (P) stratified by BMI change category (five categories) listed for each model.

All logistic regression models compared to reference category of weight stable (change in BMI (≥-1 to ≤1 kg/m^2^))

*adjusted for maternal age, sex of the infant, maternal ethnicity, maternal highest educational achievement, maternal employment status, co-morbidities, pregnancy complications, maternal smoking status, previous PB and fertility treatment, all at second pregnancy. Also adjusted for, interpregnancy interval, BMI at first pregnancy booking appointment and gestational age at first pregnancy booking appointment.

Significant P values in bold italics

**Insufficient data to run the model in underweight women whose BMI decreased by > 3 kg/m^2^.

**Table 4:** Associations of weight change between second and third live-birth pregnancies with PB at third pregnancy, stratified by BMI group at the start of the second pregnancy, in a population-based cohort from Southampton, UK 2003–2018.

Model	BMI group at first pregnancy (n)	Decrease in BMI (<-3 units)			Decrease in BMI (<-1 to ≥ -3 units)			Increase in BMI (>1 to ≤3 units)			Increase in BMI (>3 units)		
		OR	95% CI	P value	OR	95% CI	P value	OR	95% CI	P value	OR	95% CI	P value
**Unadjusted**	**All women (5,108)**	1.58	0.87–2.85	0.130	1.05	0.67–1.66	0.832	1.14	0.80–1.63	0.456	1.19	0.81–1.76	0.379
	Underweight (168)**	-	-	-	-	-	-	0.91	0.25–3.30	0.888	0.95	0.17–5.23	0.957
	**Normal weight (2,710)**	2.43	0.55–10.67	0.241	1.03	0.54–2.00	0.916	1.34	0.85–2.11	0.205	1.39	0.82–2.35	0.217
	**Overweight (1,286)**	1.30	0.42–4.00	0.645	1.35	0.62–2.97	0.453	0.55	0.22–1.36	0.195	1.39	0.66–2.93	0.393
	**Obese (935)**	1.88	0.67–5.32	0.232	0.89	0.25–3.09	0.850	1.48	0.55–3.96	0.437	0.68	0.21–2.17	0.514
Fully-adjusted*	**All women (5,057)**	***2*.*01***	***1*.*05–3*.*87***	***0*.*035***	1.12	0.70–1.81	0.635	1.25	0.86–1.82	0.234	1.22	0.80–1.87	0.357
	Underweight (165)**	-	-	-	-	-	-	0.90	0.22–3.64	0.881	1.13	0.16–7.91	0.899
	**Normal weight (2,684)**	3.01	0.59–15.30	0.184	1.08	0.54–2.18	0.821	1.41	0.87–2.30	0.163	1.34	0.75–2.39	0.331
	**Overweight (1,255)**	1.45	0.45–4.70	0.535	1.58	0.68–3.66	0.290	0.67	0.26–1.71	0.402	1.55	0.68–3.53	0.292
	**Obese (898)**	2.06	0.66–6.41	0.212	1.11	0.30–4.14	0.882	1.96	0.67–5.72	0.220	0.55	0.15–2.06	0.371

Odds ratios (OR), confidence intervals (CI) and P value (P) stratified by BMI change category (five categories) listed for each model.

All logistic regression models compared to reference category of weight stable (No change in BMI (≥-1 to ≤1 kg/m^2^))

*adjusted for maternal age, sex of the infant, maternal ethnicity, maternal highest educational achievement, maternal employment status, co-morbidities, pregnancy complications, maternal smoking status, previous PB and fertility treatment, all at third pregnancy. Also adjusted for, interpregnancy interval, BMI at booking for second pregnancy and gestation at booking for second pregnancy.

Significant P values in italics

**Insufficient data to run the model in underweight women whose BMI decreased.

Subgroup analysis was conducted, stratifying by BMI category at the start of the initial pregnancy. In the primary cohort, the odds of PB in normal weight women who lost weight (>3 kg/m^2^) was 3.5 times higher than normal weight women who were weight stable, after adjusting for all covariates (unadjusted OR 4.0. 95% CI 2.15–7.58, aOR 3.50, 95% CI: 1.78–6.88; p<0.001) (Table 3). There were no significant findings when undertaking the same analysis on the secondary cohort (Table 4).

There were 183 women who moved from a normal weight category in their first pregnancy to an underweight category in their second pregnancy (with variable levels of weight loss depending on their starting point). Amongst these women, 6.0% of live births were premature (11 PBs). In addition, there were 110 women who were normal weight at first pregnancy and lost more than 3 kg/m^2^ by booking of their second pregnancy (although not necessarily moving to an underweight BMI depending on their starting point). Amongst these women, 10.9% of live deliveries were premature (12 PBs). Of the 110 women, 27 moved from a normal weight at their first pregnancy booking to underweight at their second pregnancy booking. In this group there were 6 PBs, at a rate of 22%. Amongst the remaining 83 women, who lost more than 3 kg/m^2^ but remained normal weight for both pregnancies, 7.8% of live deliveries were premature (6 PBs).

Subgroup analysis was also conducted by the type of PB. Spontaneous PBs were analysed by excluding indicated premature deliveries and comparing with term deliveries. In the primary cohort (n = 427 spontaneous PB, n = 14,447 term), weight loss (>3 kg/m^2^) in women who were normal weight at first pregnancy was associated with increased odds of spontaneous PB (unadjusted OR 4.03, 95%CI 2.03–7.97, aOR 3.34, 95% CI 1.60–6.98, p = 0.001). There were no significant findings in the secondary cohort.

Indicated PBs were analysed by excluding spontaneous PB. In the primary cohort (n = 87 indicated PB), substantial weight gain (>3 kg/m^2^) in normal weight women increased odds of indicated PB (unadjusted OR 2.30, 95% CI 1.15–4.58, p = 0.018). This association was attenuated after adjusting for confounders (aOR 1.84, 95% CI 0.86–3.95, p = 0.115). There were no significant findings in the secondary cohort for the univariable models and the subgroup sample was insufficient to run the fully-adjusted models.

## Discussion

This study aimed to examine the association between maternal interpregnancy weight change and PB, in an English population-based cohort. Just under half of women gained weight (>1 kg/m^2^) between their first and second, or second and third pregnancies. There were fewer indicated than spontaneous PBs in both cohorts (16.9% and 18.4% of PBs were indicated in the primary and secondary cohort respectively), which is broadly consistent with other European cohorts of singleton pregnancies [55]. Women who had PBs were more likely to be smokers and to have a comorbidity, and were less likely to have a university degree. Interpregnancy weight loss between the first and second pregnancy (primary cohort) in normal weight women was associated with PB. This was also the case when analysing the whole sample of women with second to third pregnancy data (secondary), but not in the stratified analysis by BMI category group at the start of the second pregnancy. There was no association between interpregnancy weight gain and PB after adjusting for confounders.

Some studies also found weight loss in normal weight women to be associated with PB [12, 22]. While others found no association between interpregnancy weight loss and PB [15, 23, 56] but all of these studies had small sample sizes and may have been underpowered to detect differences, given the relatively low event rate for PB. Additionally, these studies failed to adjust for some key confounders such as maternal comorbidities, previous PB and infant sex.

It is unclear from the literature why interpregnancy weight change may be associated with PB [12]; there are several potential mechanisms. It is possible that the associations seen are not true associations, but are instead markers for unmeasured confounding. For example, psychosocial stress and underlying poor mental and physical health may all be contributing to both weight loss [18] and PB [17]. In the primary cohort, normal weight women who had lost weight were significantly more likely to have a shorter interpregnancy interval, be younger and be smokers than normal weight women who were weight stable or gained weight (data not shown). They also tended to be less likely to have a university degree, or be employed, both markers of socioeconomic status that may be indicative of increased stress, although neither of these differences were statistically significant.

Some women who were normal weight and lost weight moved into the underweight BMI range (n = 183 in the primary cohort). Having an underweight BMI in itself is a risk factor for PB [10, 44], and again the underlying mechanisms are unclear, but possibly due to micro- and macronutrient malnutrition [44, 57, 58]. The increased risk in some of these women may be a result of becoming underweight at second pregnancy, rather than the change in weight. Similarly, those who lost large amounts of weight in a short space of time, or who became underweight, might be malnourished, increasing their chances of infection during pregnancy (another risk factor) [16, 17].

There was a marginally increased risk of PB in women who lost >3 kg/m^2^ between second and third pregnancies in the whole sample, but not in the stratified baseline BMI subgroup analyses. Wallace et al [15], the only other group to consider second to third pregnancy did not replicate this finding. It is worth noting that Wallace et al [15] did not adjust for several key confounders (including previous PB, underlying co-morbidities and pregnancy complications), which is likely to have affected their results.

In this study, there was no evidence of association between weight gain and PB in adjusted models. However, Riley et al [59] reported that weight gain is associated with a decreased risk of spontaneous PB, in those who were normal weight or overweight at first pregnancy. Wallace et al [13] found that weight gain (≥3 kg/m^2^) was associated with reduced risk in all women, although this became non-significant when only including those women with a BMI of 25 or less at first pregnancy. Weight gain (>3 kg/m^2^) in normal weight women between first and second pregnancies was associated with indicated PB in an unadjusted model. This finding became non-significant after accounting for confounders. Villamor and Cnattingius [12] also found an increased risk of indicated PB among normal or overweight women who gained weight, which became non-significant after removing women with comorbidities from the analysis. McBain et al [11] also report that interpregnancy weight gain in overweight or obese women is associated with PB, but they did not adjust for maternal comorbidities in the second pregnancy.

### Strengths and limitations

The study had numerous strengths. Utilising routine antenatal healthcare data for all births reduces the risk of selection bias as the cohort represents the regional population, and recall bias as exposure information was collected prospectively. This study had a relatively large sample size, compared with some of the other studies [11, 13, 15, 23, 56, 60, 61] in the literature. This study used USS dating rather than LMP wherever possible to ensure the most accurate gestation measurements, and adjusted for the majority of known confounders. Additionally, this study is only the second to consider second to third pregnancy [15], and so adds to the body of evidence for this group.

It is likely that the secondary cohort was underpowered to detect differences in PB in subgroup analyses. This is because the effect size is expected to be relatively small and PB is a reasonably uncommon occurrence, so a large cohort is required in order to detect differences. This issue is exacerbated when considering spontaneous and indicated PB separately, particularly indicated PB, which made up just 0.58% of the births in the primary cohort (0.76% in the secondary cohort). This may explain why many of the statistically significant findings in the primary cohort were non-significant in the secondary cohort.

The final dataset had a lower percentage of live-born PBs than might be expected (5.0% in the first pregnancy and 3.4% in the second pregnancy in the primary cohort, and 3.9% in the second pregnancy and 4.2% in the third pregnancy in the secondary cohort). This is comparable with Scottish rates of 5.8% [62] but less than the UK average of 7.8% [63]. The dataset as a whole had a rate of 7.2%, but this percentage dropped when exclusions were applied (Fig 1), with the largest reductions being seen as multiple pregnancies, which are strongly associated with PB [10], were removed.

Limitations from using routine data collection include missing data, difficulty ensuring consistency of measurement techniques and equipment, and having to make use of the information that is collected. This meant that some variables were not available, such as gestational weight gain, breastfeeding duration and lifestyle changes such as changes in diet and exercise habits. Where possible the data were checked for consistency and variables that were not considered valid were either entered as missing or omitted from the analysis. Maternal height was self-reported which is a limitation, although this was mitigated (as far as possible) by excluding women whose height was not consistent between the initial and subsequent pregnancy. After applying all exclusions, the percentage excluded in the primary cohort was 15.8%, and in the secondary cohort 15.1% (Fig 1). Additionally, information on preterm pre-labour rupture of membranes (PPROM) was not available, so any women with PPROM who had an induction to augment labour will have been classified as indicated rather than spontaneous PB. This limits the reliability of the sub-analyses on indicated PB.

Due to multiple statistical testing, the study is vulnerable to an increased chance of type one error. In order to reduce the risk of this, a more stringent statistical significance level of p ≤ 0.01 could be applied. At this level, weight loss (>3 kg/m^2^) in normal weight women between first and second pregnancy would remain significantly associated with increased PB. The association between weight loss and PB in the overall sample in the secondary cohort would not remain statistically significant using this threshold.

### Implications for research and practice

The interpregnancy period is a key opportunity for optimising health before a subsequent pregnancy [64], with the intensive health professional contact provided after birth. Given the known adverse outcomes associated with obesity during pregnancy [65], current advice is for obese women to lose weight before conceiving [66]. This study found no evidence that weight loss in obese women was associated with increased risk of PB. Given the association between weight loss in normal weight women and PB, and the links between being underweight and PB [44], and other poor outcomes [67]^,^[68], perhaps advice around weight change should also be given for normal and underweight women. Encouraging normal weight women to remain weight stable may help to reduce PB, as well as other poor maternal and neonatal outcomes [69]. Encouraging underweight women to eat a balanced diet may result in a normal BMI, reduced nutrient deficiency and better outcomes. However, any recommendations made would have to consider the wider context of other maternal and neonatal outcomes [70], as well as the practicalities involved in supporting new mothers to make lifestyle changes, at a time when they have already had significant life changes and their own health may be less of a priority than that of their child [71]. Supporting women who are unsupported or dealing with challenging circumstances may be particularly important, given the possible associations between stress, socio-economic status, poor maternal health and PB [37–39, 45].

## Conclusion

In this study, interpregnancy weight loss for women who had normal weight in their first pregnancy was associated with increased risk of PB in their second pregnancy, but not in overweight or obese women. Interpregnancy weight gain was not associated with PB. Current national guidelines encouraging obese women to lose weight before a pregnancy should be followed to reduce other maternal and offspring adverse outcomes of maternal obesity. The interpregnancy period is a key window of opportunity for preventative input to improve maternal health and thus outcomes.
